# Effect of Humidity on Light-Activated NO and NO_2_ Gas Sensing by Hybrid Materials

**DOI:** 10.3390/nano10050915

**Published:** 2020-05-09

**Authors:** Abulkosim Nasriddinov, Marina Rumyantseva, Elizaveta Konstantinova, Artem Marikutsa, Sergey Tokarev, Polina Yaltseva, Olga Fedorova, Alexander Gaskov

**Affiliations:** 1 Chemistry Department, Moscow State University, 119991 Moscow, Russia; naf_1994@mail.ru (A.N.); artem.marikutsa@gmail.com (A.M.); pergeybokarev@gmail.com (S.T.); yal-polina@yandex.ru (P.Y.); fedorova@ineos.ac.ru (O.F.); gaskov@inorg.chem.msu.ru (A.G.); 2 Faculty of Materials Science, Moscow State University, 119991 Moscow, Russia; 3 Physics Department, Moscow State University, 119991 Moscow, Russia; liza35@mail.ru; 4 Faculty of nano-, bio-, information and cognitive technologies, Moscow Institute of Physics and Technology, Dolgoprudny, 141700 Moscow Region, Russia; 5 National Research Center “Kurchatov Institute”, 123182 Moscow, Russia; 6 A.N. Nesmeyanov Institute of Organoelement Compounds RAS, 119991 Moscow, Russia

**Keywords:** organic–inorganic hybrid materials, tin dioxide, indium oxide, Ru(II) complex, nitrogen monoxide NO, nitrogen dioxide NO_2_, semiconductor gas sensor, humidity effect

## Abstract

Air humidity is one of the main factors affecting the characteristics of semiconductor gas sensors, especially at low measurement temperatures. In this work we analyzed the influence of relative humidity on sensor properties of the hybrid materials based on the nanocrystalline SnO_2_ and In_2_O_3_ and Ru (II) heterocyclic complex and verified the possibility of using such materials for NO (0.25–4.0 ppm) and NO_2_ (0.05–1.0 ppm) detection in high humidity conditions (relative humidity (RH) = 20%, 40%, 65%, 90%) at room temperature during periodic blue (λ_max_ = 470 nm) illumination. To reveal the reasons for the different influence of humidity on the sensors’ sensitivity when detecting NO and NO_2_, electron paramagnetic resonance (EPR) spectroscopy and diffuse reflectance infrared Fourier transform spectroscopy (DRIFTS) investigations were undertaken. It was established that the substitution of adsorbed oxygen by water molecules causes the decrease in sensor response to NO in humid air. The influence of humidity on the interaction of sensitive materials with NO_2_ is determined by the following factors: the increase in charge carrier’s concentration, the decrease in the number of active sites capable of interacting with gases, and possible substitution of chemisorbed oxygen with NO_2_*^−^* groups.

## 1. Introduction

Nitric oxide (NO) and nitrogen dioxide (NO_2_) gases produced by fuel combustion, electric power plant boilers and industrial plants [1,2] pose a serious problem for both the environment and human health [3,4]. On the other hand, several investigations showed that nitric oxide in exhaled samples of breath condensate can indicate lung diseases and respiratory tract inflammation [5,6,7,8]. An early exhaled NO test will enable the correct diagnosis to guide therapy and prevent progression of the disease. The difficulty of quantifying NO in exhaled air is due to the low level of its concentration (20–200 ppb) against the background of a wide range of interfering impurities (CO, CO_2_, acetone, etc.), as well as high relative humidity (RH) in the range of 90–100%.

Currently, the most reliable method for quantitative analysis of NO is based on the reaction between NO and ozone O_3_, accompanied by chemiluminescence, the intensity of which is proportional to the NO content in the sample. Chemiluminescence occurs in the infrared (IR) range when excited electrons in the NO_2_ molecule pass to lower energy levels.

Direct determination of nitric oxide in the gas phase is possible using semiconductor gas sensors. The advantages of gas sensors in comparison with methods of chemiluminescent analysis, mass spectrometry, gas chromatography, etc. are the ability to obtain results in real time without sample processing, small size, low power consumption, relatively low price. The analysis of the exhaled air can be performed directly by the patient without the participation of specially trained personnel. Therefore, the sensors used for this application have to be stable in ambient humidity, sensitive and selective to NO in the ppb range.

The humidity of the environment is one of the main factors affecting the efficiency, device lifetime and long-term stability of semiconductor gas sensors. The influence of water vapor on the characteristics of semiconductor gas sensors becomes especially significant at low temperatures, and this is most evident at room temperature. Most studies on the sensor properties of semiconductor metal oxides to NO gas in ambient humidity confirm the deterioration of the main sensor characteristics: response and recovery times, sensor signal, and sensitivity much more than toward NO_2_. Moreover, in some cases, the enhancement of sensor signal is observed [9,10,11,12,13]. These results indicate the complex nature of the interaction of target gases with semiconductor oxide materials in the presence of water vapor. Further and deeper investigation of the gas-sensing mechanism in humid air, especially at room temperature, could prevent and avoid its negative impact.

Our previous studies [14,15] demonstrated high sensitivity and selectivity of photosensitized organic–inorganic hybrid materials toward NO and NO_2_ detection at room temperature under visible light activation. In this work, we analyzed the influence of relative humidity on sensor properties of the hybrid materials based on nanocrystalline SnO_2_ and In_2_O_3_ and Ru (II) heterocyclic complex as photosensitizer and verified the possibility of using such materials for NO and NO_2_ detection in high ambient humidity conditions at room temperature during periodic blue light-emitting diode (LED, λ_max_ = 470 nm) illumination.

## 2. Materials and Methods

Nanocrystalline SnO_2_ and In_2_O_3_ were synthesized by the chemical precipitation method and a heteroleptic Ru(II) complex was used as a photosensitizer. A detailed scheme of their synthesis is given in our previous work [14]. Hybrid materials were prepared by adsorption of Ru (II) complex on the surface of semiconductor oxides.

The composition and microstructure of the materials obtained were investigated by X-ray diffraction (XRD), Raman spectroscopy, scanning electron microscopy (SEM), high-resolution transmission electron microscopy (HRTEM), energy-dispersive X-ray (EDX) spectroscopy, X-ray photoelectron spectroscopy (XPS), and Brunauer–Emmett–Teller (BET) measurements; the optical properties and thermal stability were studied via ultraviolet/visible (UV/Vis) absorption spectroscopy and thermogravimetric analysis (TGA), respectively, and were discussed in detail previously [14]. Surface spin centers were studied by electron paramagnetic resonance (EPR) spectroscopy. The EPR spectra were recorded at 110 K to reduce thermally induced signal broadening by the Bruker ELEXSYS−580 spectrometer (X-band, sensitivity is ~10^10^ spin/G, Bruker, Billerica, MA, USA). The values of g-factors and spin center concentrations were calculated based on Mn^2+^ and CuCl_2_·2H_2_O standard samples, respectively. Additionally, SnO_2_ sample modified with Ru was used in order to understand the influence of the Ru on concentration of the surface radicals. Ruthenium acetylacetonate (Sigma-Aldrich, >97%, St. Louis, MO, USA) with a concentration of 1% (weight) per metal was used as a precursor. Chemical modification of nanocrystalline SnO_2_ was carried out by impregnation with the alcohol solution of Ru(acac)_3_. The obtained powder was annealed for 24 h at the lowest temperature necessary for decomposition of precursor (T = 265 °C).

Samples characteristics (composition, microstructure parameters, photoresponse in dry pure air) are summarized in Table 1.

In order to study the reactivity of the hybrid materials in an atmosphere of NO and NO_2_, in situ diffuse reflectance infrared Fourier transform spectroscopy (DRIFTS) was carried out on a Perkin-Elmer Spectrum One Fourier Transform Infrared spectrometer (Perkin Elmer Inc., Beaconsfield, UK) at room temperature. The spectra were registered in the range of 4000–1000 cm^−1^ with a resolution of 4 cm^−1^ and accumulation of 30 scans under a controlled gas flow rate of 100 mL/min. The samples were preheated at 50 °C for 1 h and cooled down to 25 °C in dry air. The gas mixtures containing 5 ppm of NO_2_ or 50 ppm of NO were prepared by dilution of certified gas mixtures 10 ± 1 ppm of NO_2_ in N_2_ or 100 ± 5 ppm of NO in N_2_, respectively (Monitoring, St. Petersburg, Russia). The purified air from a pure air generator (Granat-Engineering Co. Ltd., Moscow, Russia) was used as background and carrier gas.

For gas sensor measurements, the sensitive materials were deposited on specially designed dielectric micro-hotplates (Al_2_O_3_), covered with Pt contact electrodes for resistance measurements and with a Pt heater. The square-shape micro-hotplates with dimensions of 0.9 × 0.9 × 0.15 mm were fully covered by the samples. The thickness of the films, estimated from the preliminary calibration carried out by scanning electron microscopy, was about 1 µm. Direct current DC measurements have been carried out at room temperature under constant flux of 100 mL/min NO/air (0.25–4.0 ppm NO in dry air) or NO_2_/air (0.05–1.0 ppm NO_2_ in dry air) gas mixtures in dry and humid air (RH = 20–90%) under periodic blue (λ_max_ = 470 nm) light illumination. In this procedure the illumination of the sensor element is carried out in a pulsed mode with a short period (2 min “on”–2 min “off”). The steady state can be characterized by the minimum resistance *R_light_*, which is achieved during the sensor illumination, and the maximum *R_dark_*, which is achieved in the dark period. The resistive response can be calculated as the ratio *S* = *R_dark gas_*/*R_dark air_* of dark resistances (measured at the end of 2 min “light off” period) at a given NO_x_ concentration *R_dark gas_* and in pure air *R_dark air_*. A relative humidity both in DRIFTS and sensor measurements was set and controlled by Humidifier P-2 (Cellkraft AB, Sweden). A detailed description of the sensor fabrication and measurements setup can be found in our previous works [14,15].

## 3. Results and Discussion

In this study the gas sensor measurements of the obtained materials were performed when detecting NO (0.25–4.0 ppm) and NO_2_ (0.05–1.0 ppm) in dry and humid atmosphere (RH = 20%, 40%, 65%, 90%) at room temperature. The samples were illuminated with a blue LED (λ_max_ = 470 nm) in pulsed mode during 2 hours under dry air or in humid air (depending on the type of measurements) before the first measurement to reach a stable resistance value, and irradiation was kept during the whole experiment in gas phase.

**Figure 1:**
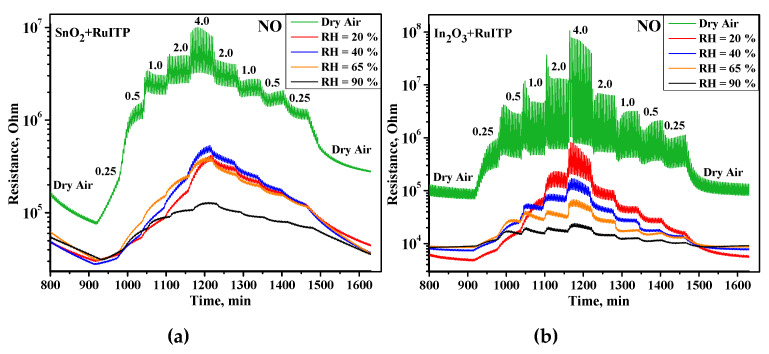
Room temperature electrical resistance change of the (**a**) SnO_2_+RuITP and (**b**) In_2_O_3_+RuITP hybrid materials under periodic illumination during stepwise increase and decrease of the NO concentration in dry air and humid air.

Electrical resistance measurements are plotted in Figure 1a,b only for the SnO_2_+RuITP and In_2_O_3_+RuITP hybrid materials, respectively, since all samples show a similar trend in the presence of both NO and NO_2_ in a humid atmosphere: resistance increases with stepwise increasing concentration of introduced gases, which is typical for *n*-type semiconductors in the presence of oxidizing gases. With an increase in the concentration of NO and NO_2_, a slow and partial accumulation of adsorbates on the surface of materials occurs, and with a decrease in concentration it takes longer for the resistance to reach its initial stationary state, since photodesorption of residual adsorbates proceeds slowly. Therefore, the resistance values for the same concentrations with a stepwise increase and decrease in concentration do not coincide. In the ideal case they should be equal, but due to the kinetic inhibition of the processes at room temperature, they differ slightly at low concentrations. The same phenomenon may also be observed even in the case of gas sensors operating during dynamic thermal heating [16].

The interaction of *n*-type semiconductor oxides with NO_2_ in dry air accompanied by a decrease in electrical conductivity can be described with following reactions:

NO_2(gas)_ = NO_2(ads)_ (1)

NO_2(gas)_ + *e*^−^ = NO_2_^−^_(ads)_ (2)

2 NO_2(gas)_ + ½ O_2(gas)_ + 2 *e*^−^ = NO_2_^−^_(ads)_ + NO_3_^−^_(ads)_ (3)

NO_2_^−^_(ads)_ + ½ O_2(gas)_ = NO_3_^−^_(ads)_ (4)

At the same time NO sensing in dry air is determined by the oxidation with chemisorbed oxygen:

NO_(gas)_ + ½ O_2(gas)_ + *e*^−^ = NO_2_^−^_(ads)_. (5)

**Figure 2:**
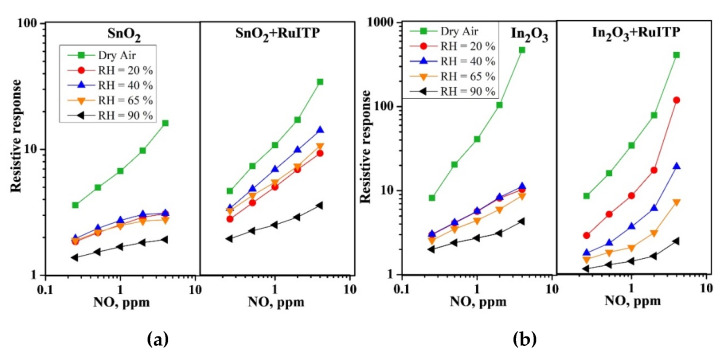
Resistive response of (**a**) SnO_2_, SnO_2_+RuITP and (**b**) In_2_O_3_, In_2_O_3_+RuITP samples depending on NO concentration in dry and humid air at room temperature.

**Figure 3:**
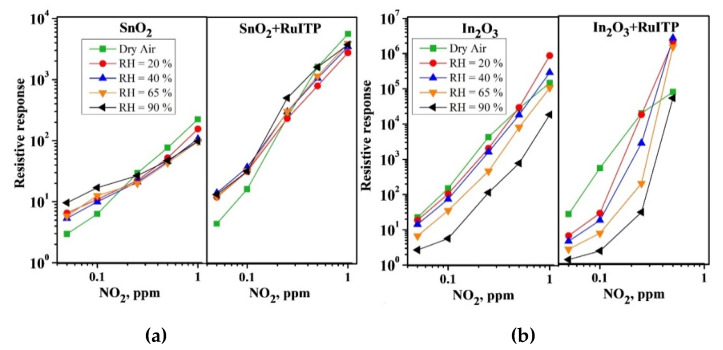
Resistive response of (**a**) SnO_2_, SnO_2_+RuITP and (**b**) In_2_O_3_, In_2_O_3_+RuITP samples depending on NO_2_ concentration in dry and humid air at room temperature.

As discussed in [14], the interaction of NO and NO_2_ with the surface of semiconductor oxides occurs in a similar way, through the mechanism of adsorption with the localization of electrons of the semiconductor conduction band. The lower sensitivity of hybrid materials to NO (Figure 2), compared to NO_2_ (Figure 3), should be due to different initial steps in the detection routes. In reaction with NO_2_, it is a simple one-electron reduction (reaction (2)) favored by the strong oxidative activity of nitrogen dioxide. The interaction with NO (reaction (5)) is essentially an oxidation of the target gas mediated by oxygen on the surface of the semiconductor oxide. This should be the main reason for the different sensitivities to NO_2_ and NO, although the surface species produced in both interaction pathways are similar. Figure 1, Figure 2 and Figure 3 show that an increase in relative humidity in the range 0–90% leads to a decrease in the baseline resistance of all materials, as well as to a change in the sensor signal when interacting with nitrogen oxides.

The main trends in a change in the resistive response to NO and NO_2_ depending on air humidity can be summarized as follows:

(i) When detecting NO for all samples with an increase in air humidity there is a decrease in the resistive response (Figure 2a,b);

(ii) For SnO_2_-based samples, when detecting low NO_2_ concentrations (C(NO_2_) < 0.25 ppm), the resistive response increases with RH (Figure 3a,b);

(iii) For In_2_O_3_ based samples, with increasing humidity there is an increase in the resistive response when detecting high concentrations of NO_2_ (C(NO_2_) ≥ 0.5 ppm). Note that for C(NO_2_) = 1 ppm, the value of the dark resistance of In_2_O_3_+RuITP hybrid sample exceeds the upper measurement limit of the device used (10^10^ Ohm). Therefore, the point corresponding to C(NO_2_) = 1 ppm for In_2_O_3_+RuITP hybrid sample is not shown in Figure 3.

The main contribution to the electrophysical properties of semiconductor gas sensors at room temperature in air is mainly made by adsorbed oxygen and water molecules. Temperature has a significant influence on the predominant form of chemisorbed oxygen, as well as the concentration of adsorbed oxygen and hydroxyl groups on the surface of semiconductor oxides, which determined the width of an electron-depleted layer near the surface of crystal grains of the semiconductor [17,18]. It was shown [19] that water is desorbed at temperatures above 100 °C, O_2_ molecules—at temperatures above 250 °C, OH groups are removed when heated above 500 °C from the surface of SnO_2_ films. Moreover, the density of adsorbed oxygen atoms increases in the temperature range from 200 °C to 500 °C, and then begins to decrease (desorption predominates). This means that molecular oxygen, surface water and OH groups, on the one hand, and chemisorbed forms of oxygen, on the other hand, compete for the same active sites on the surface of metal oxides. At low operating temperatures at the surface of *n*-type semiconductor metal oxides, the dissociative adsorption of H_2_O is energetically more preferable than dissociative adsorption of oxygen and surface becomes hydroxylated [20,21]. Moreover, different types of chemisorbed oxygen ions can exist at different temperature ranges: molecular (O_2_^−^) is formed at temperature lower than 150 °C and atomic (O^−^, O^2−^)—at higher temperatures [17,19,22]. That is why in our experiments at room temperature in air flow we can assume that a depletion layer at the surface of SnO_2_ and In_2_O_3_ is mainly formed by O_2_^−^ species.

There are several mechanisms explaining the increase in conductivity in humid air described in the literature [17,21]:

(i) During dissociative adsorption of H_2_O isolated hydroxyl groups are formed due to the acid-base interaction of OH with the lattice Sn (Lewis acid). The released hydrogen atom reacts with the lattice or chemisorbed oxygen (Lewis base) with the formation of rooted hydroxyl group and injection of an electron into the conduction band [23,24]:

H_2_O_(gas)_ + Sn_(lat)_ + O_(lat)_ = [Sn_(lat)_–OH] + [O_(lat)_H]∙+ *e*^−^ (6)

(ii) Another mechanism involves the formation of the OH groups, which bind to the Sn atom, and ionization of the oxygen vacancy, which provides additional electrons:

H_2_O_(gas)_ + 2Sn_(lat)_ + O_(lat)_ = 2[Sn_(lat)_–OH] + V_O_∙+ 2*e*^−^ (7)

(iii) The electron affinity of the acceptor surface states can change after interaction with OH^−^ or H^+^. Also chemisorbed oxygen ions can be replaced or rearranged by the water molecules [25,26]:

H_2_O_(gas)_ + 2Sn_(lat)_ + O_2_^−^_(ads)_ = 2[Sn_(lat)_–OH] + *e*^−^ (8)

**Figure 4:**
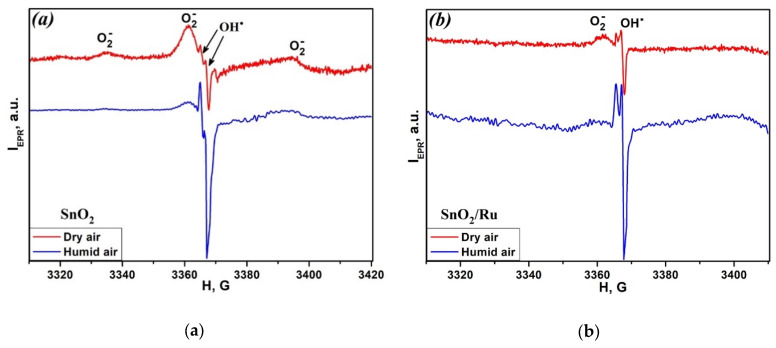
Electron paramagnetic resonance (EPR) spectra of SnO_2_ (**a**) and SnO_2_/Ru (**b**) in dry air and after exposure to humid air.

To find out the reasons for the different influence of humidity on the sensor sensitivity of SnO_2_ and In_2_O_3_ semiconductor oxides and hybrid samples when detecting NO and NO_2_, EPR and DRIFTS investigations were undertaken. The influence of the relative humidity on the concentration of the surface radicals was investigated by EPR spectroscopy. Figure 4a,b shows the EPR spectra of SnO_2_ and SnO_2_, modified with Ru. The EPR signal with an intense central line (*g* = 2.0030 ± 0.0005) and weak lateral satellites (*g* = 2.0210 ± 0.0005 and *g* = 1.9833 ± 0.0005), according to literature [27,28], belong to O_2_^−^ radicals.

An EPR signal with parameters g = 2.0021 ± 0.0005 and g = 2.0009 ± 0.0005 for tin dioxide based materials is not described in the literature. However, it is known that for TiO_2_ sample the EPR signal with close g-factors belongs to OH∙ radicals [27,29,30]. The additional EPR measurements were carried out for samples differing in the degree of surface hydration to check out the hypothesis of the formation of hydroxyl radicals on the surface of SnO_2_. Hydrated samples were obtained by passing saturated water vapor over SnO_2_ and SnO_2_/Ru powders during 24 h. It can be seen from Figure 4a,b that the EPR spectra of samples processed by this method demonstrate an increase in signal intensity and concentration with g = 2.0021 ± 0.0005 and g = 2.0009 ± 0.0005. Thus, the nature of this signal is not in doubt and corresponds to OH∙ radicals. Note that the intensity of the EPR signal from O_2_^−^ radicals (and, accordingly, their concentration) in hydrated samples decreased. The concentrations of O_2_^−^ and OH∙ radicals on the surface of SnO_2_ and SnO_2_/Ru samples in dry and humid conditions presented in Table 2 indicate that O_2_^−^ radicals can be easily replaced by OH∙ radicals in a humid atmosphere. Changes in the ratio of OH∙/O_2_^−^ concentrations in dry and humid air for SnO_2_ and SnO_2_/Ru are close and are 6.5 and 6.3, respectively.

**Figure 5:**
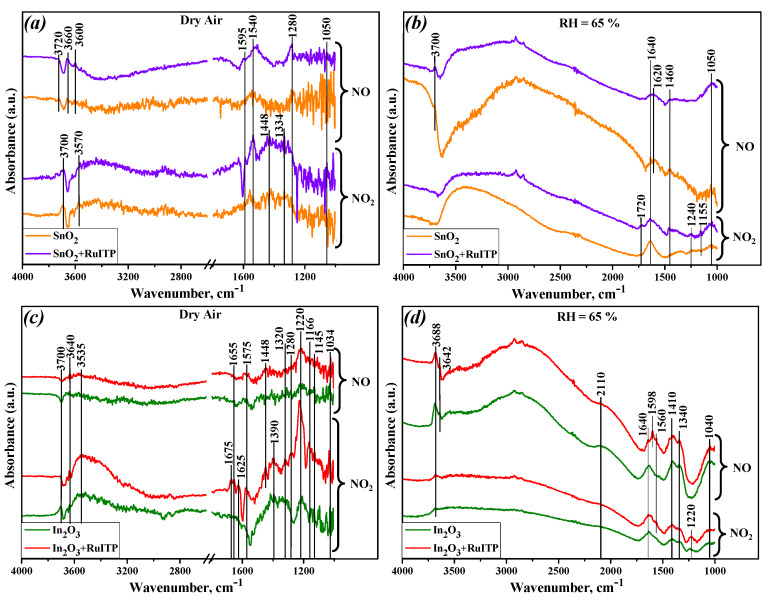
In situ diffuse reflectance infrared Fourier transform spectroscopy (DRIFT) spectra of the nanocrystalline SnO_2_ and hybrid SnO_2_+RuITP samples in dry (**a**) and humid (RH = 65%) air (**b**); nanocrystalline In_2_O_3_ and hybrid In_2_O_3_+RuITP samples in dry (**c**) and humid (RH = 65%) air (**d**) after 60 min exposure of the NO (50 ppm) and NO_2_ (5 ppm) at room temperature.

Figure 5 shows the DRIFT spectra of the nanocrystalline SnO_2_ and In_2_O_3_ and hybrid materials after 1 h interaction with NO (50 ppm) and NO_2_ (5 ppm) in dry and humid air (RH = 65%) at room temperature. There are a lot of IR bands in dry air comparing with the humid atmosphere. Moreover, the appearance of nearly identical absorption IR bands corresponding to NO_x_^−^ groups upon adsorption of NO and NO_2_ indicates an almost similar interaction nature of these two molecules with the surface of SnO_2_ and In_2_O_3_ semiconductors. Recently, the same results were obtained for nanocrystalline WO_3_ with different particle size by the authors of [31] and for SnO_2_-based sensors in [32].

The bands assignments observed upon interaction of NO and hybrid samples have been fully discussed in our previous paper [14]. Additional bands observed during interaction of NO_2_ with analyzing samples in dry and humid air are summarized in Table S1 (Supplementary Information)  [32,33,34,35].

The most intense bands at the 1145–1205 cm^−1^ and 1205–1220 cm^−1^ regions correspond to chelating and bridging bidentate NO_2_^−^ groups. The nitrate species NO_3_^−^ can be assumed at the wide range of frequency: 970–1040, 1180–1600 and 1260–1300 cm^−1^. The bands between 1605 and 1680 cm^−1^ are attributed to adsorbed NO_2_, which can be overlapped with bending H_2_O vibration modes. Adsorbed NO_2_ molecules were obtained during adsorption of NO, NO+O_2_ or NO_2_ on the surface of different catalysts and metal oxides [36,37]. It is difficult for some peaks to exactly distinguish and determine the structure type of the adsorbed species, because different type of nitrites and nitrates have IR-activated bonds in the same wavenumber region.

For In_2_O_3_ and its hybrid sample, a broad band appears at 2110 cm^−1^ in a humid atmosphere in the presence of NO. This band has also been detected by other researchers [38] and has been assigned either to nitrosonium ion NO^+^ or nitronium ion NO_2_^+^. However, most authors tend to assume that during the adsorption of NO/O_2_ in the presence of moisture this band is attributed to NO^+^ ion, which produces with the participation of the acidic hydroxyls, but NO_2_^+^ often arise after NO_2_ adsorption [39,40]. Moreover, the appearance of this band correlates with the disappearance of the OH stretching band at 3610 cm^−1^. There is an additional band at 1720 cm^−1^ appearing upon NO_2_ adsorption in humid air on the surface of SnO_2_+RuITP hybrid sample. This band is attributed to dinitrogen tetraoxide N_2_O_4_, which can be easily formed by NO_2_ dimerization [41].

The high wavenumber region is characterized by surface hydroxyl groups. The spectra in O-H stretching (3200–3700 cm^−1^) and H–O–H bending (1610–1640 cm^−1^) regions look similar for all samples. The negative sharp bands produced in these regions are associated with the interaction and/or replacement of hydroxyls by adsorbed NO*_x_*^−^ species. The background of this frequency range of the spectrum increased in a humid atmosphere due to the adsorption of a large number of water molecules.

In dry gas phase the hybrid materials have additional and more intense bands than pure nanocrystalline metal oxides. Moreover, NO_2_ adsorption compared to NO adsorption leads to an increase in the intensity of bands associated with nitrite and nitrate species. This effect can be explained through the following assumptions: 

(i) Sensitization of SnO_2_ and In_2_O_3_ semiconductors with a heterocyclic Ru (II) complex leads to an increase in the number of charge carriers (electrons) in the conduction band of semiconductor matrix, which enhance the interaction with the electrophylic NO_2_ molecules;

(ii) NO_2_ as a strong oxidizing gas will either capture electrons in direct competition with a greater advantage than oxygen, since its electron affinity is 5 times greater (E_ea_(NO_2_) = 2.27 eV, E_ea_ (O_2_) = 0.44 eV) and increases the sensor resistance; or react with the surface chemisorbed oxygen ions, or can replace it in the competitive adsorption (reactions 2 and 3);

(iii) The electron affinity of nitrogen monoxide (E_ea_(NO) = 0.03 eV) is lower than that of both NO_2_ and O_2_ [42]. There is an unpaired electron on the antibonding 2π orbital of the NO molecule, thus it is difficult for it to accept an electron or replace the oxygen. That’s why there wasn’t appear any bands attributed to nitrosyl anion (NO^−^) or NO dimers in NO adsorption spectra (Figure 5). On the other hand, the formation of nitrate and nitrite groups after the interaction of materials with NO can be associated with the oxidation of nitrogen monoxide molecules by chemisorbed oxygen on the surface of semiconductor oxides [31]. G.X. et al. have investigated the NO adsorption and oxidation over various SnO_2_ surfaces by density functional theory (DFT) calculations [43]. It was concluded that NO molecules are mainly adsorbed on the SnO_2−*x*_ (110) surface containing pre-adsorbed O_2_ and oxidize to form NO_2_^−^ species.

In a humid atmosphere a notable difference among spectra can be found: the band at 1220 cm^−1^ for In_2_O_3_ and In_2_O_3_+RuITP samples (Figure 5d), that corresponds to a bidentate nitrite group, has appeared only after NO_2_ exposure and was not observed in the presence of NO. This band is less intense and shifted to the 1240 cm^−1^ position for SnO_2_ and SnO_2_+RuITP samples (Figure 5b). Considering the above position and the results of EPR studies, we can safely assume that in a humid atmosphere hydroxyl groups replace chemisorbed oxygen molecules, which play a key role in the oxidation of NO molecules. As a result, no absorption bands corresponding to bidentate nitrite groups are observed. Previously, Sergent et al. [44] have been investigated the influence of the different nitrite and nitrate species on the electrical response of nanocrystalline SnO_2_ and TiO_2_ powders and established that it is bidentate nitrate species that are responsible for the change in conductivity of SnO_2_ and TiO_2_ and sensor response to NO_2_. Thus, for successful detection of low NO concentrations in a humid atmosphere, additional modification of the sensitive material is necessary, for example, by introducing an additional layer that acts as a filter to absorb water molecules, or by introducing an additional catalyst that ensures the oxidation of NO to NO_2_.

## 4. Conclusions

The analysis of data obtained by EPR and DRIFTS methods, together with the dependence of the resistive response of samples in the presence of NO and NO_2_ in dry and humid air, allows us to reach the following conclusions:

(i) When detecting NO, a decrease in the resistive response is observed with an increase in air humidity for all samples. This is due to a decrease in the number of adsorbed NO_2_^−^ particles on the surface of materials (according to DRIFTS). The reason for this, in turn, is the substitution of adsorbed oxygen by water molecules (according to EPR data), which makes it difficult to oxidize NO by reaction (5). For successful detection of low NO concentrations in a humid atmosphere, additional modification of the sensitive material is necessary.

(ii) The influence of humidity on the interaction of sensitive materials with NO_2_ is complex. It can be concluded that for SnO_2_-based samples, an increase in the electron concentration in accordance with the reactions (6–8) stimulates reaction (2), which leads to an increase in the sensor response in the region of low NO_2_ concentrations. When the NO_2_ concentration increases, the limiting factor is the number of active sites capable of interacting with gases, which decreases with increasing humidity. For In_2_O_3_-based samples, a decrease in the sensor response to low NO_2_ concentrations with increasing humidity indicates that an increase in the concentration of charge carriers in accordance with the reactions (6)–(8) is not a determining factor. At the same time, the dependence of the sensor response of In_2_O_3_-based samples to high NO_2_ concentrations on air humidity is non-monotonic. The maximum response value is observed at RH = 20% and RH = 40%. It can be assumed that under these conditions, the reaction of substitution of chemisorbed oxygen with NO_2_^−^ groups makes an additional contribution to the formation of the sensor response:

NO_2(gas)_ + O_2_^−^_(ads)_ = NO_2_^−^_(ads)_ + O_2(gas)_ (9)

Due to the significant difference in the electron affinities O_2_ and NO_2_ molecules, at high NO_2_ concentration the equilibrium (9) will be shifted to the right, and chemisorbed NO_2_^−^ species should form deeper acceptor levels in the band gap of In_2_O_3_ that will increase the resistance of In_2_O_3_ based samples.

## Figures and Tables

**Table 1:** Sensor characteristics of synthesized materials.

Sample	Phase Composition	d_XRD_^1^, nm	d_TEM_^2^, nm	S_surf_^3^, m^2^/g	Average Pore Diameter, nm	$\frac{\left[Ru\right]}{[Ru]+{\left[M\right]}^{4}}$, *at*. %	R_av_^5^, Ohm Pure Air	S_Ph_^6^, in Pure Air (λ = 470 nm)
SnO_2_	SnO_2_, cassiterite In_2_O_3_, bixbyite	4 ± 1	4 ± 1	115 ± 5	3–5; 70–80	-	7.8·10^4^	1.00
SnO_2_+RuITP		7 ± 1	7 ± 2	90 ± 5	3–4	1.2 ± 0.1	6.9·10^5^	1.22
In_2_O_3_						-	1.7·10^4^	1.25
In_2_O_3_+RuITP						2.1 ± 0.2	5.8·10^5^	1.95

^1^ crystallite size from X-ray diffraction (XRD); ^2^ particle size from transmission electron microscopy (TEM); ^3^ specific surface area; ^4^ obtained by energy-dispersive X-ray spectroscopy (EDX) on thick films: M = Sn for SnO_2_+RuITP sample; M = In for In_2_O_3_+RuITP sample; ^5^ resistances; ^6^ effective photoresponse.

**Table 2:** Concentrations (spin/g) of O_2_^−^ and OH∙ radicals on the surface of SnO_2_ and SnO_2_/Ru samples in dry and humid conditions.

Conditions	SnO_2_			SnO_2_/Ru		
	O_2_^−^	OH∙	OH∙/O_2_^−^	O_2_^−^	OH∙	OH∙/O_2_^−^
Dry air	8 × 10^14^	1.9 × 10^14^	0.24	2.5 × 10^14^	4.4 × 10^14^	1.2
Humid air	2.8 × 10^14^	4.3 × 10^14^	1.54	10^14^	1.1 × 10^15^	11

## Supplementary Materials

The following are available online at https://www.mdpi.com/2079-4991/10/5/915/s1: Table S1: Frequency ranges and structures of IR bands observed in DRFIT spectra in 5 ppm NO_2_/air and 50 ppm NO/air atmosphere on the surface of prepared samples in dry and humid (RH = 65%) air at room temperature.
