# Supplementary material for: Effect of Humidity on Light-Activated NO and NO2 Gas Sensing by Hybrid Materials
Source: Nanomaterials (Basel). 2020 May 9;10(5):915. doi: 10.3390/nano10050915 (PMC7279420; doi:10.3390/nano10050915)
Supplement: Supplementary file 1 [file nanomaterials-10-00915-s001.pdf]

Effect of humidity on light activated NO and NO_2 gas sensing by hybrid materials

Abulkosim Nasriddinov [1,2], Marina Rumyantseva [1*], Elizaveta Konstantinova [3,4,5], Artem Marikutsa [1], Sergey Tokarev [1,6], Polina Yaltseva[1], Olga Fedorova [1,6] and Alexander Gaskov [1]

1 Chemistry Department, Moscow State University, Moscow, 119991 Russia; E-mails: roum@inorg.chem.msu.ru (M. R.), naf_1994@mail.ru (A.N.), artem.marikutsa@gmail.com (A.M.), yal-polina@yandex.ru (P.Ya.), gaskov@inorg.chem.msu.ru (A.G.)
2 Faculty of Materials Science, Moscow State University, Moscow, 119991 Russia
3 Physics Department, Moscow State University, 119991 Moscow, Russia; liza35@mail.ru
4 Faculty of nano-, bio-, information and cognitive technologies, Moscow Institute of Physics and Technology, Dolgoprudny, Moscow Region, 141700 Russia
5 National Research Center "Kurchatov Institute", Moscow, 123182 Russia
6 A.N. Nesmeyanov Institute of Organoelement Compounds RAS, Moscow 119991 Russia; pergeybokarev@gmail.com (S.T.), fedorova@ineos.ac.ru (O.F.)

Supplementary Information

Table S1. Frequency ranges and structures of IR bands observed in DRFIT spectra in 5 ppm NO_2/air and 50 ppm NO/air atmosphere on the surface of prepared samples in dry and humid (RH = 65 %) air at room temperature according to [1 - 4].

Structure	Assignments	Frequency range, cm^{-1}
M – O – N – O	monodentate nitrite	1065 – 1210; 1400 – 1485;
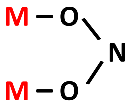	bridging bidentate nitrite	1205 – 1220; 1470 – 1520;
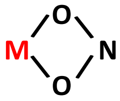	chelating bidentate nitrite	1145 – 1205; 1266 – 1314;
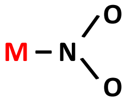	nitro compound	1250 – 1350; 1370 – 1470;
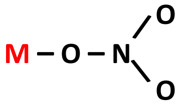	monodentate nitrate	970 – 1035; 1250 – 1290; 1480 – 1540;
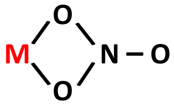	chelating bidentate nitrate	1010 – 1040; 1260 – 1300; 1550 – 1600;
	adsorbed NO_2	1605 – 1642; 1673 – 1680;
H – O – H	bending H_2O	1610 – 1640;
O H ··· O H	H-bound rooted OH groups	3200 – 3550;
M – O – H	"free" or isolated OH groups	3600 – 3700.

M = Sn or In.

References

1. Leblanc, E.; Perier-Camby, L.; Thomas, G.; Gibert, R.; Primet, M.; Gelin, P. NO_x adsorption onto dehydroxylated or hydroxylated tin dioxide surface. Application to SnO_2-based sensors. *Sensors and Actuators B* **2000**, *62*, 67 – 72.
2. Hadjiivanov, K.I. Identification of neutral and charged N_xO_y surface species by IR spectroscopy. *Catal. Rev. Sci. Eng*. **2000**, *42*, 71–144.
3. Nakamoto, K. Infrared and Raman spectra of inorganic and coordination compounds. Part A: theory and applications in inorganic chemistry, 6th ed.; John Wiley & Sons, Inc.: New Jersey, USA, 2009.
4. Socrates, G. *Infrared and Raman characteristic group frequencies: tables and charts*, 3rd ed.; John Wiley & Sons Ltd: West Sussex, England, 2001.
